# In-Depth Characterization of Bioactive Extracts from *Posidonia oceanica* Waste Biomass

**DOI:** 10.3390/md17070409

**Published:** 2019-07-09

**Authors:** Isaac Benito-González, Amparo López-Rubio, Antonio Martínez-Abad, Ana-Rosa Ballester, Irene Falcó, Luis González-Candelas, Gloria Sánchez, Jesús Lozano-Sánchez, Isabel Borrás-Linares, Antonio Segura-Carretero, Marta Martínez-Sanz

**Affiliations:** 1 Food Safety and Preservation Department, IATA-CSIC, Calle Catedrático Agustín Escardino Benlloch 7, Paterna, 46980 Valencia, Spain; 2 Department of Analytical Chemistry, Nutrition and Food Sciences, University of Alicante, San Vicente del Raspeig, 03690 Alicante, Spain; 3 Food Biotechnology Department, IATA-CSIC, Calle Catedrático Agustín Escardino Benlloch 7, Paterna, 46980 Valencia, Spain; 4 Microbiology and Ecology Department, University of Valencia. Avda. Dr. Moliner, 50. Burjassot, 46100 Valencia, Spain; 5 Center of Research and Development of Functional Food. Health Science Technological Park, Avda. del Conocimiento s/n, 18100 Granada, Spain; 6 Department of Food Science and Nutrition, University of Granada, Campus Universitario s/n, 18071 Granada, Spain; 7 Department of Analytical Chemistry, Faculty of Sciences, University of Granada, 18071 Granada, Spain

**Keywords:** valorisation, antiviral, antifungal activity, antioxidant capacity, ultrasound, hot water extraction

## Abstract

*Posidonia oceanica* waste biomass has been valorised to produce extracts by means of different methodologies and their bioactive properties have been evaluated. Water-based extracts were produced using ultrasound-assisted and hot water methods and classified according to their ethanol-affinity (E1: ethanol soluble; E2: non-soluble). Moreover, a conventional protocol with organic solvents was applied, yielding E3 extracts. Compositional and structural characterization confirmed that while E1 and E3 extracts were mainly composed of minerals and lipids, respectively, E2 extracts were a mixture of minerals, proteins and carbohydrates. All the extracts showed remarkably high antioxidant capacity, which was not only related to phenolic compounds but also to the presence of proteins and polysaccharides. All E2 and E3 extracts inhibited the growth of several foodborne fungi, while only E3 extracts decreased substantially the infectivity of feline calicivirus and murine norovirus. These results show the potential of *P. oceanica* waste biomass for the production of bioactive extracts.

## 1. Introduction

Bioactive compounds with antioxidant and antimicrobial activity have found applications in a broad range of areas, like biomedicine or food science, and thus prospection of new biomass resources to obtain extracts with these functional properties is a topic of great interest [1,2]. Specifically in the food science area, certain microorganisms can negatively affect the quality, safety and shelf-life of food products. Furthermore, oxidative processes can have an impact on food quality promoting, for instance, rancidity. These oxidative processes are also known to affect human health due to free radical formation leading to changes in protective enzymes, membrane lipids or even DNA; a fact which has been correlated to the development of several diseases. 

With regards to the raw materials used for the production of bioactive-rich extracts, although medicinal plants have been typically used up to date [3], the circular economy policies currently being promoted in Europe, aiming to reduce and reuse the different types of residues, are fostering the exploration of alternative biomass resources [4,5,6]. In particular, aquatic biomass, such as seaweeds and aquatic plants, represent an interesting alternative for the production of bioactive extracts, given their great availability and abundance and their high content in bioactive components, such as sulphated polysaccharides, polyphenols and fatty acids [7]. *Posidonia oceanica (P. oceanica)* is a marine plant endemic to the Mediterranean Sea which forms extensive meadows, preventing erosion and hosting many marine species. However, during its lifecycle the leaves detach off their stems and accumulate on the coasts, generating a residue which is detrimental to the quality of the beaches and whose elimination generates significant expenses to the local authorities. Bioactive extracts from *P. oceanica* with antioxidant and/or antidiabetic potential have been reported [8,9], but these were produced using organic solvents, with associated environmental issues and not desirable for food applications, and made use of the native plant, extracted from its natural habitat, instead of the residue. 

In contrast, the use of innocuous solvents, such as water, has been very limited and very few works have reported on the production of water-extracted bioactive materials [10]. On the other hand, greener methodologies, which avoid the use of organic and/or toxic solvents, reduce the emission of contaminants to the environment and minimize the amount of processing steps and processing time, are currently being explored. The potential of greener methodologies such as enzyme-assisted, microwave-assisted and pressurised-liquid extractions for the production of bioactive molecules from marine sources has been reported in several works [11,12,13]. In particular, the potential of ultrasound (US)-based treatments has been evaluated as they can reduce both time and the energy input required (lower temperatures are needed) while maintaining the yield [12,14]. Moreover, US capability of disrupting cell walls benefits mass transfer and thus, promotes the extraction of bioactive components with lower solvent ratio [14]. On the other hand, hot water treatments have been reported to efficiently extract different carbohydrates such as rhamnose, xylose or glucose, as well as proteins from different marine seaweed species [14]. Despite both the extraction medium and methodology used are expected to affect the composition and functional properties of plant-based extracts, complete characterizations of the extracts are barely carried out [8,15]. This combined compositional and functional characterization is required in order to optimize the extraction protocols and make a proper correlation between composition and functional properties. In fact, most of the existing literature only relates the antioxidant and/or antimicrobial activity capacity of extracts with their total phenolic content [3,15,16,17,18], although it is known that other compounds, such as polysaccharides and peptides, can also show a high bioactive potential [19,20].

In this work, the main objective was to exploit *P. oceanica* waste biomass as a potential source of bioactive extracts. In addition to the conventional organic solvent extraction protocol, alternative greener water-based extraction protocols based on heat and ultrasound-assisted methods were explored for the production of extracts. An exhaustive analysis of the composition and bioactive properties (antioxidant, antifungal and antiviral capacity) of the generated extracts was carried out with the aim of evaluating their potential as bioactive food or pharma ingredients, as well as extending the knowledge on the direct impact of the extraction method on the properties of the extracts. 

## 2. Results and Discussion

### 2.1. Composition and Structural Characterization of the P. oceanica Extracts

*P. oceanica* biomass extracts were produced by using water-based and organic solvent-based extraction protocols. Both the nature of the solvent and the extraction method used were expected to result in extracts with different composition and properties [11,12]. The yields for the water-based extractions, calculated with respect to the initial dry weight of *P. oceanica* biomass, were 6.0 ± 0.8% for E1 US, 9.3 ± 2.8% for E1 H_2_O, 2.1 ± 0.1% for E2 US and 3.0 ± 0.3% for E2 H_2_O. These results were very similar to those obtained by applying ultrasound and hot water extraction techniques to raw biomass from *Lentinus edodes* [14]. Interestingly, the ultrasound treatment allowed to decrease 4-fold the extraction time with respect to the heating treatment, but the extraction yields were not significantly (*p* > 0.05) reduced, hence highlighting its potential as an alternative cost-effective and energy-saving method. The organic solvent-based extractions gave rise to yields of 10.8 ± 2.2% for E3 US and 9.4 ± 2.8% for E3 H_2_O, which were higher than the values previously reported for *P. oceanica* extracts obtained with organic solvents using ultrasound and NaOH pre-treatments (~1.0–7.5%) [21]. The differences in the yields from the E1, E2 and E3 extracts are expected to arise from their distinct composition and the relative abundance of their components in the raw *P. oceanica* biomass.

The composition of the extracts was quantitatively evaluated and the results are compiled in Table 1. As deduced from the results, the E1, E2 and E3 extracts presented very different compositions. While the E1 extracts were mainly constituted of ashes (72–75%), the E2 extracts contained relatively high fractions of ashes (34–41%), proteins (27–37%) and carbohydrates (12–29%). On the other hand, and as already anticipated, the E3 extracts, obtained using organic solvents, were mainly composed of lipids (56–79%). It is worth noting that for the water-based extractions, the ultrasound treatment led to higher amount of extracted ashes, whereas greater amounts of carbohydrates, proteins and polyphenols were extracted by the heating treatment. Although ultrasound techniques have been previously reported to effectively disrupt the cell wall structure in aquatic biomass materials, aiding the extraction of less accessible components [22], the reduced extraction time applied in this case was most likely responsible for the lower quantities of extracted components. On the other hand, despite the reduced extraction time, the cell wall disruption produced by the ultrasound treatment seemed to promote a greater extraction of lipids in E3 US as compared to E3 H_2_O.

Phenolic compounds are one of the most relevant components targeted in plant extracts, since their abundance is usually correlated with high antioxidant capacity values [15]. Although organic solvents are commonly preferred to produce polyphenol-rich extracts, glycosylated polyphenols are expected to be more easily extracted with water [23]. This may explain the fact that the E2 H_2_O extract presented the highest total phenolic content (~9%) from all the samples, followed by the E3 extracts (~8%). The total phenolic contents in these extracts were lower when compared with those from extracts from *P. oceanica* leaves where free and bound phenolic compounds were concentrated by standard but more complex methodologies. For instance, a procedure consisting of an ultrasound treatment (90 min) using a mixture of methanol–water (80:20 *v*/*v*), followed by hydrolysis with NaOH and extraction with ethyl acetate, was reported for the extraction of free and bound phenolic compounds (328–407 mg GAE/g extract, respectively) [21]. Thus, it seems that longer or more disruptive methods are required for the release of phenolic compounds which may be bound to proteins and/or polysaccharides in the cell walls of *P. oceanica* biomass. Different phenolic compounds have been identified in *P. oceanica* leaves by HPLC, being chicoric acid the most abundant one and also having significant amounts of gentisic acid and ferulic acid [24]. These phenolic compounds might play a significant role in the bioactivity of the obtained extracts. 

**Figure 1:**
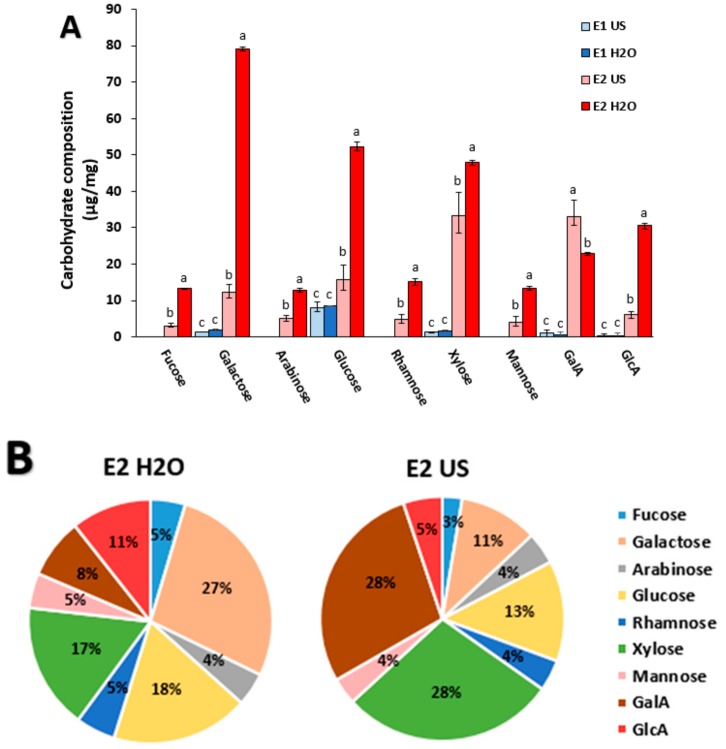
Carbohydrate composition of the aqueous *P. oceanica* extracts. (**A**) Absolute concentration values for E1 and E2 extracts and (**B**) their relative abundance in the E2 extracts. Different letters denote significant monosaccharide content differences between extracts (*p* ≤ 0.05).

Apart from the phenolic compounds, the bioactive properties of extracts from aquatic biomass have also been attributed to the presence of polysaccharides [7]. Given their potential interest, a compositional carbohydrate analysis was carried out in E1 and E2 extracts (as E3 did not contain significant amounts of carbohydrates (cf. Table 1). As observed in Figure 1A, the low carbohydrate content in the E1 extracts (~1 wt%) can only be attributed to low molecular weight oligosaccharides or monosaccharides, since these extracts corresponded to the fraction that did not precipitate upon ethanol addition. The composition was rather similar for E1 H_2_O and E1 US, glucose being the main sugar component (~0.8 wt%), followed by very minor quantities of xylose and galactose. These minor oligo- or monosaccharides might be present as part of metabolic (in the case of glucose) or cell wall synthesis processes, or even arise from minor polymer degradation during the extraction process. The E2 extracts contained the ethanol precipitated polysaccharides. In this case the E2 US and E2 H_2_O extracts differed in their quantitative and qualitative composition. As shown in Figure 1B, the E2 H_2_O extract showed higher contents in most sugar units, especially galactose, as well as glucose, whereas the E2 US extract was comparatively enriched in galacturonic acid and xylose. Similar results have been obtained by applying microwave extractions to *P. oceanica* leaves in terms of galactose and glucose contents [13]. Given the mild extraction conditions (neutral pH and temperature <100 °C), cellulose or tightly bonded hemicelluloses were not likely to be extracted [25,26]. Therefore, we speculate that the differences might be associated to pectin extraction (galacturonans in the case of E2 US and galactans in E2 H_2_O extracts), while loosely or more soluble hemicelluloses (mainly glucans and xylans), might also be extracted to some extent. The more efficient extraction under heat treatment (E2 H_2_O) would therefore yield higher pectin content, in form of galactans and arabinogalactans, as the probable major pectin component in Posidonia species [27]. The preferential extraction of galacturonans with the ultrasonic treatment, at the expense of lower extraction yields, is in line with previously published results [22]. Glucuronoarabinoxylans are the main hemicellulose component in herbaceous plants and seem to be equally extracted with both techniques, while glucan extraction would be favoured with the heating treatment [28]. To the best of our knowledge, there is no previous literature related to the polysaccharide composition of *P. oceanica*. A more detailed compositional analysis of the raw material is out of the scope of this article and may constitute the subject of future research.

**Figure 2:**
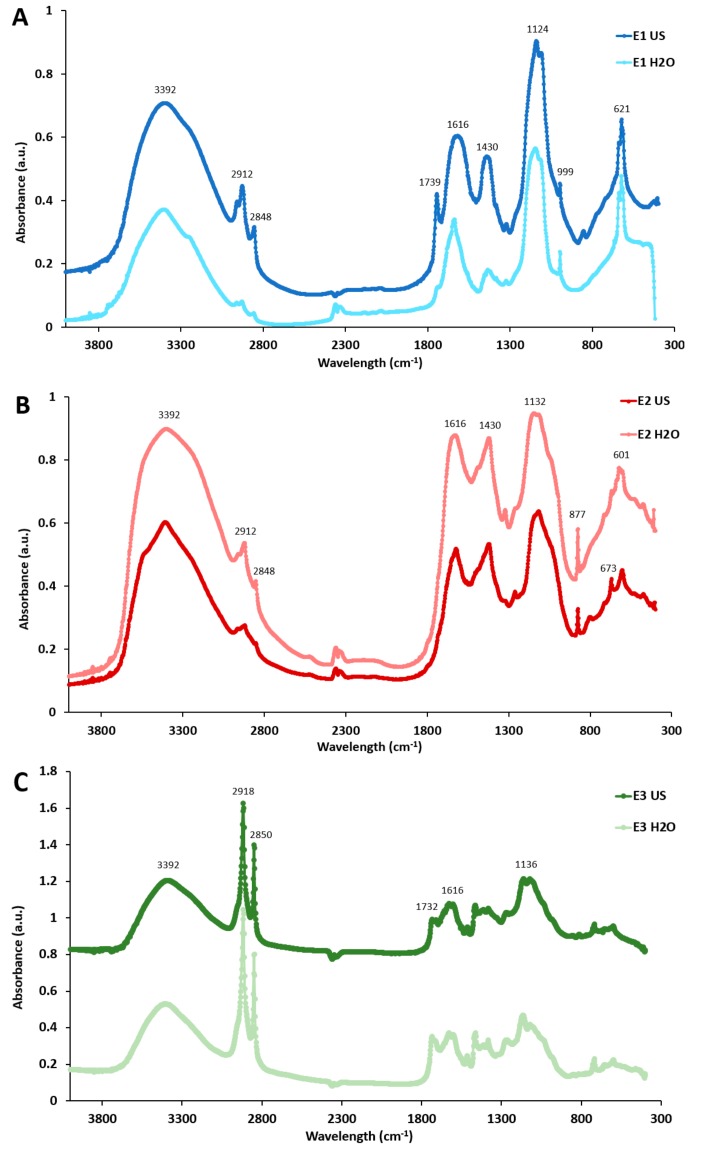
FT-IR spectra of the different *P. oceanica* extracts obtained by means of water-based extractions (**A**,**B**) and organic solvent-based extractions (**C**). E1 US, E2 US and E3 US spectra have been offset for clarity.

FT-IR analyses were carried out to confirm the compositional differences in the *P. oceanica* extracts. The obtained spectra, shown in Figure 2, evidence the marked compositional differences between the E1 and E2 extracts (water-based extractions) and the E3 extracts (organic solvent-based extractions), with the former ones showing several characteristic bands corresponding to proteins, polyphenols and polysaccharides, while characteristic bands from lipidic components were detected in the spectra from the latter ones. The FT-IR spectra of E1 and E2 extracts contained signals at around 3392, 2912, 2848, 1616, 1430 and 1124 cm^−1^. The broad band observed around 3392 cm^−1^ is assigned to the stretching vibrations of the O−H groups present in phenolic compounds and polysaccharides as well as N−H stretching vibrations from proteins. The small bands located at 2912 and 2848 cm^−1^ are attributed to symmetric and asymmetric C−H, CH_2_ and CH_3_ stretching and bending vibrations. The peak observed at ~1618 cm^−1^ is characteristic of protein amides, whereas the region located at 1200 to 600 cm^−1^ can be associated to polysaccharide characteristic peaks, ascribed to C−C and C−O stretching modes. The aforementioned bands could not be unequivocally attributed to the presence of proteins, polysaccharides and polyphenols in the extracts, as they have also been reported to arise from the presence of mineral compounds [29]. 

According to the compositional analysis (cf. Table 1), the E1 extracts were mainly composed of ashes, i.e. mineral compounds. The FT-IR spectra from these samples were very similar to those from a low combusted ash sample of eastern Spain Mediterranean biomass (containing *Pinus halepensis*, *Quercus coccifera* and *Rosmarinus officinalis*) [30]. Furthermore, the band located between 1080 and 1180 cm^−1^ has been previously attributed to the stretching vibration of Si−O groups from the inorganic components in native soil samples (ash content > 80%) [31] and was present in both E1 extracts, in agreement with the high ash content detected for these extracts (cf. Table 1). Furthermore, the appearance of a sharp and intense peak at 621 cm^−1^, which has been previously ascribed to different compounds such as sulphur, phosphorous or silica [32] confirmed the high inorganic matter content in the E1 extracts. 

Although the spectra from the E2 extracts showed some similarities with the E1 extracts (due to their high mineral content), the shape of the most intense peaks differed significantly, most probably due to the overlapping of mineral characteristic peaks with bands arising from the presence of polysaccharides and proteins in the E2 extracts. This is the case of the 1700 to 1400 cm^−1^ region, where two intense peaks at around 1616 and 1430 cm^−1^ (typically associated to the primary amide and CH_2_ and CH_3_ asymmetric deformation of proteins, respectively) had a sharper appearance in the case of the E2 extracts. Several peaks were detected for the E2 extracts within the polysaccharide “fingerprint” region (i.e., 1200–800 cm^−1^). For instance, the intense and sharp peak detected at around 877 cm^−1^ in both E2 extracts is known to correspond to the pyranose rings from mannose and galactose [33] whose content was remarkably high, especially in the case of E2 H_2_O. Additionally, the peaks located at ~673 and 601 cm^−1^, which appeared sharper in the E2 US extract, have been previously assigned to the presence of xylan-type polysaccharides [34], supporting the greater xylose content in E2 US as evidenced by the carbohydrate compositional analysis (Figure 1B).

Finally, in agreement with the high lipidic content of the E3 extracts, two sharp and intense peaks were detected at 2918 and 2850 cm^−1^, corresponding to the CH_2_ asymmetrical and symmetrical stretching from fatty acids. Additional peaks arising from the presence of lipids were also detected at ~1730 cm^−1^ (C==O of ester groups), 1616 cm^−1^ (carboxylic group) and 1136 cm^−1^ (C−O). It should be noted that *P. oceanica* has been characterized by the presence of long-chain fatty acids (C_22_−C_34_), such as palmitic, palmitoleic, oleic and linoleic acids [35].

**Figure 3:**
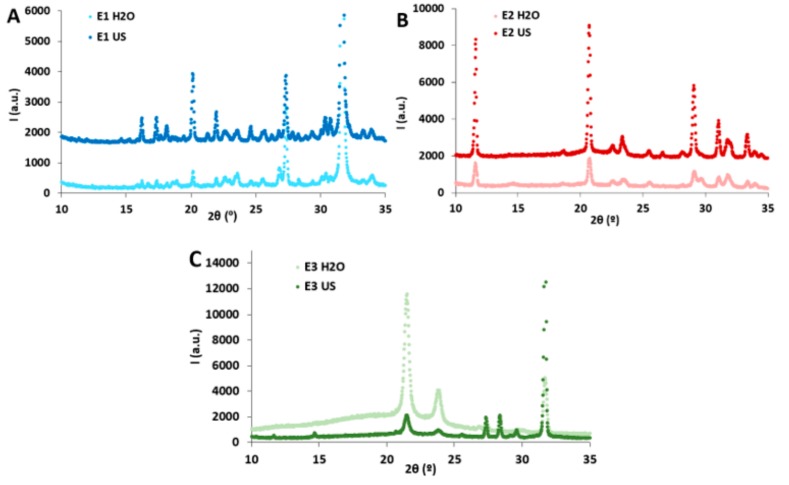
XRD patterns of the freeze-dried extracts. (**A**,**B**) Water-soluble extracts and (**C**) organic-soluble extracts. The spectra from E1 US and E2 US have been offset for clarity.

The X-ray diffraction patterns of the different extracts are shown in Figure 3. As observed, both E1 extracts showed three intense peaks at 20°, 27° and 32°. These diffraction peaks have been previously related to the presence of mineral compounds. The peak at 20° has been assigned to the presence of silicates [36], the peak at 27° has been attributed to calcite in ash samples from lignocellulosic biomass [37,38] and the peak at 32° has been attributed to serendibite in seaweed ash biomass [36]. Moreover, three additional peaks located at 16°, 17° and 22° were present in the case of E1 US, which have been associated to sulphate-containing minerals [38]. Most of these mineral components, particularly silicates, are responsible for maintaining the rigidity of plant tissues and are supposed to be introduced into the feedstock through endocytosis during the growth stage [37]. The E2 extracts displayed their most remarkable diffraction peaks located at 12°, 21° and 29°, being these much more intense and defined in E2 US. The peak at 12° has previously been related to crystalline structures containing galacturonic acid [39], while the peak at 21° has been reported to be present in xylanes [40]. These results are consistent with the carbohydrate composition analyses (Figure 1B), which showed greater ratios of galacturonic acid and xylose in E2 US. On the other hand, the peak at 29° has been attributed to the presence of KCl in an extract obtained from *Enteromorpha clathrata* with a protein, carbohydrate and ash composition similar to that from the E2 extracts [36].

In the case of E3 extracts, the detected diffraction peaks were expected to arise from the presence of lipids and minerals, according to their composition. While the most intense peaks in the E3 H_2_O extract were those located at 21° and 24°, the most intense peak for the E3 US extract was located at 32°. The peak at 21° has been detected in different linoleic acid polymorphs [41]. Although it was not possible to unequivocally assign these peaks to specific fatty acids, the results seem to indicate that a different lipidic profile was attained in the extracts depending on the treatment applied prior to the organic solvent extraction. 

The thermal stability of the different extracts was evaluated by means of TGA and the results are shown in Figure S1 (Supplementary Material). As observed, once again, the water-extracted E1 and E2 extracts presented a very different behaviour to that from the organic solvent-extracted E3 extracts. While the total weight loss at 600 °C was of ~20–30% and 30–50% for the E1 and E2 extracts, respectively, it was >60% for the E3 extracts. This again confirms the presence of significant amounts of thermally-resistant minerals in the E1 and E2 extracts. The peaks detected within the range of 50 to 150 °C correspond to dehydration processes and were more evident in the E1 and E2 extracts, given their more hydrophilic character. The E1 extracts did not show any evident degradation peaks within the range of 150 to 550 °C, which was most likely due to the higher thermal stability of the inorganic materials mainly composing these extracts (which typically undergo thermal degradation at 600–800 °C [30]. On the other hand, the E2 extracts presented a broad peak with the maximum located at ~260 °C, which may arise from the degradation of carbohydrates [29]. In fact, a previous study reported that the hemicelluloses and the cellulose contained in *P. oceanica* biomass show degradation processes with their maximum located at 260 °C and 334 °C, respectively [4]. A second peak, centred at ~480 °C, was also detected in E2 H_2_O. This could arise from the presence of arabinoxylan [42], arabinogalactan and galactomannan [43], which were more abundant in E2 H_2_O. 

The E3 extracts were characterized by a multiple degradation step profile, starting at temperatures higher than 220 °C. The maximum degradation rate occurred at 240 °C for E3 US, while for E3 H_2_O the maximum degradation rate corresponded to the higher temperature peak at 430 °C. This might be due to the thermal degradation of major lipidic compounds present in *P. oceanica*, such as oleic and linoleic acid, whose degradation temperatures range between 350 °C and 450 °C [44]. Moreover, the association of these lipids with more thermally stable minerals in the E3 H_2_O extract may be responsible for the greater thermal stability of this extract.

### 2.2. Evaluation of the Bioactive Properties from the P. oceanica Extracts

#### 2.2.1. Antioxidant Capacity

The antioxidant capacity of the extracts was evaluated by the ABTS and β-carotene bleaching assays and the results are gathered in Table 1. As observed, the E2 H_2_O extract presented the highest antioxidant capacity according to the ABTS method (~730 µmol TE/g extract), followed by E3 H_2_O and E2 US. Considering the results from the compositional analysis (cf. Table 1), there was not a direct correlation between the antioxidant capacity and the polyphenol content in the extracts. Although the E2 H_2_O and E3 H_2_O were within the extracts having the highest polyphenol contents, that was not the case for E2 US. It seems that, as previously noted for other water-based extracts from aquatic biomass [45], apart from polyphenols some other compounds such as proteins and polysaccharides may also contribute to the antioxidant potential of the extracts. All the extracts presented values higher than 200 µmol TE/g extract, which are comparatively greater than the extracts from other plants such as a range of medicinal Indian plants [15] (which presented an average value of 270 μmol TE/g extract) and fifteen different species of medicinal Mediterranean plants (with their water and methanolic extracts ranging from ~100 to ~1000 μmol TE/g extract) [46]. This highlights the great potential of the *P. oceanica* extracts as antioxidant agents. In particular, given its environmentally friendly character and ease of production, the E2 H_2_O extract represents an interesting material. 

In order to confirm the antioxidant capacity using a method more directly related to their application in food systems, the β-carotene bleaching inhibitory assay was carried out with the extracts tested at different concentrations (results shown in Figure S2, Supplementary Material). From this figure it can be observed that all the extracts completely inhibited the β-carotene bleaching when tested at their highest concentration (5 mg/mL). However, when tested at the lowest concentration (0.5 mg/mL), E3 H_2_O showed the highest bleaching inhibition, followed by E3 US, E2 US and E2 H_2_O (cf. Table 1). The inhibition capacity values of the E3 extracts were very similar to those reported for the methanolic extracts from *Rhodomyrtus tomentosa* (~80% inhibitory activity tested at a concentration of 0.5 mg/mL) [47], which contained a remarkable amount of polyphenols (190 mg GAE/g extract) and flavonoids (111 mg/g extract), once again confirming that other components present in the extracts also contributed to their antioxidant capacity. On the other hand, water-soluble extracts from potato peels with high polysaccharide content (similar to the E2 extracts) showed quite lower bleaching inhibitory capacity (between 30 and 40% when tested at a concentration of 10–50 mg/mL) [48]. Lower inhibition values were also reported for aqueous extracts from almond gum (30–80% when tested at a concentration of 5 mg/mL) [49] and *Arundo donax* (61–93% when tested at a concentration of 5 mg/mL) [5], again confirming the excellent antioxidant potential of *P. oceanica* extracts.

#### 2.2.2. Antifungal Activity Assays

Antifungal activity assays were carried out in order to assess the potential of the extracts to inhibit or reduce the growth of several fungi with significant incidence in the food industry. Antifungal activity was tested *in vitro* against a collection of isolates from the postharvest fungal pathogens *P. digitatum*, *P. italicum*, *P. expansum*, *B. cinerea*, *G. candidum* and *A. niger* after 3 and 6–7 days postinoculation (dpi) (Table 2 and Figure S3). No differences in fungal growth were observed by using different concentrations of the E1 extracts neither at 3 dpi nor at 7 dpi (data not shown). However, the application of both E2 US and E2 H_2_O extracts reduced the growth of *P. digitatum* and *B. cinerea* at 3 dpi, being effective against *P. digitatum* even after 7 days. The antifungal activity of E2 US was higher than that of the E2 H_2_O extract, with a 37% and 12% of reduction in the diameter of *P. digitatum* at 3 dpi, respectively. These differences might correspond to the distinct composition between E2 extracts (carbohydrates, proteins and ashes).

Among the three extraction methods, the E3 extracts were the most effective ones reducing the growth of all six postharvest pathogens assayed, being particularly effective with a reduction of 100% in the diameter of *B. cinerea* at day 3 of both E3 US and E3 H_2_O extracts. It is also important to highlight their effectiveness in reducing the sporulation of some strains, especially *A. niger* (Figure S3). The higher effectiveness of E3 US extract compared to E3 H_2_O extract may be due to their different bioactive profiles, with a higher lipidic content in E3 US (cf. Table 1). 

In comparison with other extracts obtained from marine and terrestrial plants, including medicinal plants such as rosemary, peppermint or oregano, these extracts showed significant inhibitions in the growth of *B. cinerea* at concentrations between 6 and 25%. Plants from genus Allium and Capsicum showed highest inhibitory activities after 24 and 48 h [50]. Moreover, root extracts from the medicinal plant *Arctotis arctotoides* displayed significant reductions (from 5 to 95% at the highest concentration tested corresponding to 5 mg/ML) of the growth of *P. digitatum* and *P. italicum* [51]. However, it is important to highlight that these activities correspond to organic extractions of medicinal plants, while *P. oceanica* E2 extracts have been obtained from a residue using water-based green extraction methodologies. The antifungal activity assays of the *P. oceanica* extracts suggest the capability of these extracts to prevent food contamination by fungi, augmenting the shelf-life of the products whether being incorporated in the packaging matrixes or encapsulated in safe coatings in order to be controllably released into the food environment.

#### 2.2.3. Antiviral Activity Assays

**Figure 4:**
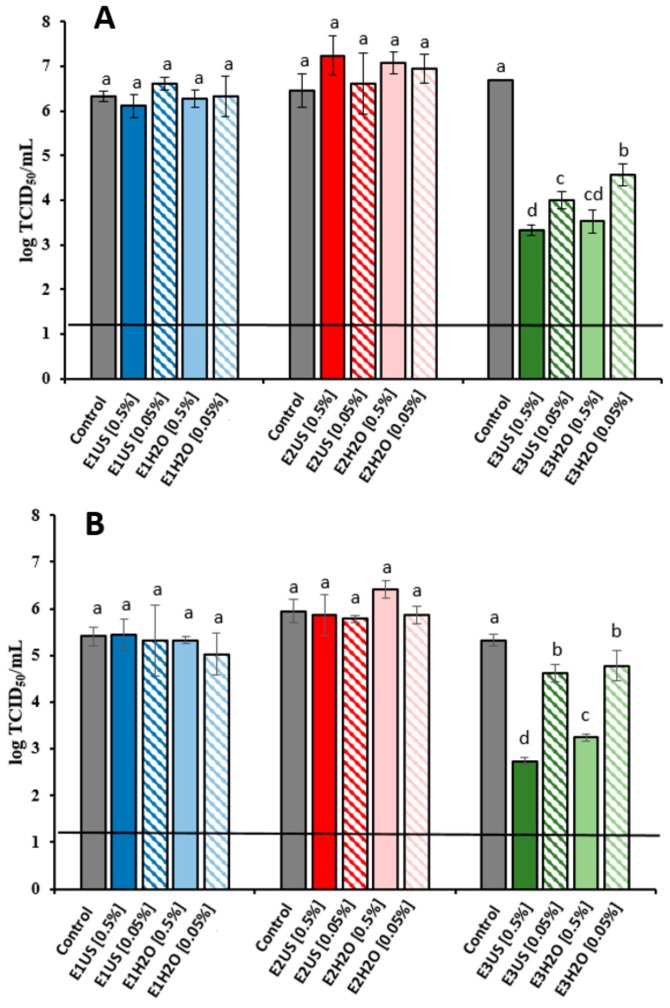
Reduction of (**A**) feline calicivirus (FCV) titres (log TCID_50_/mL) and (**B**) murine norovirus (MNV) titres (log TCID_50_/mL) treated with *P. oceanica* extracts at different concentrations (0.5 or 0.05%) after 25 °C and 37 °C ON incubations, respectively. * Each bar represents the average of triplicates. Within each column, different letters denote significant differences between treatments. ** Horizontal line depicts the detection limit.

Human noroviruses (NoVs) significantly contribute to foodborne diseases being the causative agent of one-fifth of acute gastroenteritis worldwide [52]. As propagation of NoVs is not routinely available, the infectivity of NoVs has mainly been inferred through culturable surrogates, such as feline calicivirus (FCV) and murine norovirus (MNV). Thus, in the present study the antiviral activity of the different extracts was evaluated against these two surrogates. Incubation of FCV and MNV with E1 and E2 extracts did not decrease FCV and MNV infectivity when compared to the control (Figure 4). However, results clearly show that E3 US and E3 H_2_O extracts were effective in reducing the titres of FCV and MNV in a dose-dependent manner, where increasing concentrations of E3 extracts showed increased reduction in viral titres. The antiviral activity of the E3 extracts must be therefore related to the presence of hydrophobic compounds such as non-glycosylated polyphenols and lipids. Incubation of FCV with E3 US and E3 H_2_O extracts at concentrations of 0.5% after overnight incubation at 25 °C (Figure 4A) significantly decreased FCV titres by 3.4 and 3.1 log TCID_50_/ml, respectively, while at 0.05% FCV titres were reduced by 2.7 and 2.1 log TCID_50_/ml. These results are similar to those previously reported for a range of green tea extracts obtained using different organic solvents such as ethyl acetate, methanol and hexane, when tested within the same range of concentrations than the *P. oceanica* extracts [53]. Additionally, the infectivity of a more resistant surrogate virus, MNV, was reduced by 2.6 log and 2.1 log and 0.7 and 0.5 TCID_50_/mL after treatment with E3 US and E3 H_2_O extracts at 0.5% and 0.05, respectively (Figure 4B). Similar reductions of FCV and MNV titres have been previously reported for a commercial aqueous grape seed extract tested at concentrations between 0.25 mg/mL and 1 mg/mL [54]. As the food industry is driven to seek natural alternative strategies to control foodborne pathogens E3 extracts have the potential to be used in food applications to control virus contamination. Currently, several natural compounds such as grape seed extract, green tea extract, essentials oils or their main compounds have been tested against NoV surrogates, demonstrating their potential in food applications as natural sanitizers or incorporated into biopolymers for their used as antiviral packaging or coatings (reviewed by Randazzo et al. [52]). 

## 3. Materials and Methods 

### 3.1. Materials and Reagents

Biomass waste material consisting of *Posidonia oceanica* (*P. oceanica*) leaves was collected from the shore in Calpe, Alicante (Spain) (38°38′09″N 0°04′16″E) in February–March 2017 (two different batches were collected). The collected leaves had a dark brown coloration. The material was washed vigorously with water to remove sand and salts and stored at 4 °C until its use.

2,2′-Azino-bis (3-ethylbenzothiazoline-6-sulfonic acid) diammonium salt (ABTS), (±)-6-Hydroxy-2,5,7,8-tetramethylchromane-2-carboxylic acid (Trolox) 97%, ethanol (>99.8%), toluene, acetic acid, formic acid and sodium persulphate were purchased from Sigma–Aldrich (Darmstadt, Germany). The Folin–Ciocalteau reagent, modified Lowry reagent and bovine serum albumin were obtained from the “modified Lowry protein assay kit” purchased from Thermo Fisher scientific (Madrid, Spain). All the salts, reagents and monosaccharides used for the carbohydrate composition analysis were purchased from Merck (Sigma, Darmstadt, Germany).

### 3.2. Production of P. oceanica Extracts

Different extracts were generated by treating the *P. oceanica* waste biomass to diverse extraction protocols, which are described in the following subsections. A brief description of the different extracts generated is compiled in Table 3.

#### 3.2.1. Water-Based Extractions

Water-soluble extracts were obtained by adding 100 g of wet *P. oceanica* leaves (~10 g of dry material) into 250 mL of distilled water and mixing in an electric blender until obtaining a homogeneous paste. This paste was then subjected to two different treatments: 

(i) Heating treatment: The paste was heated up to 90 °C with stirring for 2 h. After that, the material was centrifuged at 24,000 RCF and 15 °C for 20 min. The solid precipitate was separated, dried and stored at 0% relative humidity (RH) for the production of the organic-soluble extracts (see Section 2.2.2). The liquid supernatant was placed in an ice bath and the required volume of ethanol (75% with regards to the supernatant volume) was added dropwise. The material was kept stirring overnight and subsequently, it was centrifuged at 24,000 RCF and 15 °C for 20 min. The supernatant was placed in an oven at 60 °C to evaporate the ethanol and it was then freeze-dried to obtain a whitish powder extract coded as E1 H_2_O. On the other hand, the precipitate was resuspended in distilled water and freeze-dried, yielding a darker powder coded as E2 H_2_O.

(ii) Ultrasound treatment: The paste was subjected to an ultrasound treatment by immersing an ultrasound probe UP-400S (Hielscher GmbH, Hamm, Germany) operating at a maximum power of 400 W and a constant frequency of 24 kHz for 30 min. After that, the same protocol described for the E1 H_2_O and E2 H_2_O extracts was followed, obtaining the materials coded as E1 US and E2 US. The extracts were stored at 0% RH until their use.

#### 3.2.2. Organic Solvent-Based Extractions

The solid residues obtained after the first centrifugation in the heating and ultrasound treatments were subjected to a Soxhlet extraction with 800 mL of toluene:ethanol (2:1) for 24 h. The liquid phase was then collected and the solvents were evaporated by distillation on a rotary evaporator (G3 Heidolph, Schwabach, Germany) operating at 60 °C and vacuum conditions. The dry material was resuspended in 50 mL of pure ethanol, which was subsequently evaporated at room temperature to obtain the extracts coded as E3 H_2_O (obtained from the solid residue of the heating treatment) and E3 US (obtained from the solid residue of the ultrasound treatment). The extracts were stored at 0% RH until their use.

### 3.3. Chemical Composition Analysis

#### 3.3.1. Total Phenolic Content

The total phenolic content of the extracts was estimated by the Folin–Ciocalteau colorimetric assay [55]. Briefly, Folin–Ciocalteau reagent was diluted 1:10 (*v*/*v*) with distilled water and 1 mL of the final dilution was mixed with 0.2 mL of the extract sample (diluted in water, in the case of the water-soluble extracts or in ethanol, in the case of the organic-soluble extracts) at room temperature. Finally, 0.8 mL of sodium carbonate (75 mg/mL) was added and the samples were heated up to 50 °C during 30 min. Absorbance values were read at 750 nm. A calibration curve was built by using gallic acid as the standard and the total phenolic content was expressed as mg of gallic acid (GA)/g extract. All determinations were carried out in triplicate.

#### 3.3.2. Protein Content

Total protein content was measured following the Lowry method with some minor modifications [56]. Briefly, 1 mL of the modified Lowry reagent was mixed with 0.2 mL of the extracts and incubated for 10 min at room temperature. Then, 0.1 mL of Folin–Ciocalteu reagent (previously diluted 1:1 with distilled water) was added and vortexed, incubating the resulting solution for 30 min at room temperature. A blank was prepared with 0.2 mL of distilled water and the reagents and absorbance were read at 750 nm. A calibration curve was prepared with serial dilutions of bovine serum albumin (BSA) and the total protein content was expressed as mg BSA/g extract. All determinations were carried out in triplicate. 

#### 3.3.3. Lipid Content

Total lipid content was measured following the sulpho-phosphovanillin method with some minor modifications [57]. In brief, phosphovanillin reagent was prepared by dissolving vanillin in water (6 g/L) and then mixing 350 mL with 50 mL of H_2_O and 600 mL of phosphoric acid. For sample analysis, 20 µL of sample were mixed with 0.2 mL of concentrated sulphuric acid, and then stirred and incubated in boiling water for 10 min. Then, samples were conditioned with cold water during 5 min and 10 mL of phosphovanillin were added, and incubated at 37 °C during 15 min. A blank was prepared with 20 µL of ethanol and the reagents and the absorbance was finally read at 540 nm. A calibration curve was made using known concentrations of sunflower oil, and the total lipid content was expressed as mg lipids/g extract. All determinations were carried out in triplicate.

#### 3.3.4. Ash Content

Ash content was determined by the standard method TAPPI T211 om-07. Briefly, dry samples were placed in a muffle for at least 4 h at 525 ± 25 °C. Ash content was measured by the ratio of the resulting material divided by the initial weight. Determinations were carried out in duplicate. 

### 3.4. Carbohydrate Composition

The carbohydrate content and sugar composition of the extracts was determined after acidic methanolysis, as previously described [26]. The monosaccharides were analysed using high performance anion exchange chromatography with pulsed amperometric detection (HPAEC-PAD, Herisau, Switzerland) with a 940 IC system (Metrohm) equipped with a Metrosep Carb 2 column (4 × 250 mm, Metrohm, Herisau, Switzerland). Control samples of known concentrations of mixtures of glucose, fucose, galactose, arabinose, xylose, mannose, galacturonic acid and glucuronic acid were used for calibration. All experiments were carried out in triplicate.

### 3.5. Fourier-Transform Infrared Spectroscopy (FT-IR) 

Freeze-dried extract samples of ~1.2 mg were ground and dispersed in 120 mg of spectroscopic grade KBr. A pellet was then formed by compressing the sample at ~10 tons. FT-IR experiments were recorded in transmission mode in a controlled chamber at 21 °C and dry air using a Thermo Nicolet Nexus (GMI, Ramsey, MN, USA) equipment. The spectra were taken at 4 cm^−1^ resolution in a wavelength range between 400 cm^−1^ and 4000 cm^−1^ and averaging a minimum of 32 scans. 

### 3.6. X-ray Diffraction (XRD)

XRD measurements of the freeze-dried extracts were carried out on a D5005 Bruker diffractometer. The instrument was equipped with a Cu tube and a secondary monochromator. The configuration of the equipment was θ–2θ, and the samples were examined over the angular range of 3° to 60° with a step size of 0.02° and a count time of 200 s per step. 

### 3.7. Thermogravimetric Analyses (TGA)

Thermogravimetric curves (TG) were recorded with a Setaram Setsys 16/18 (SETARAM Instrumentation, Caluire-et-Cuire, France). The samples (~10 mg) were heated from 30 to 1000 °C with a heating rate of 10 °C/min under nitrogen atmosphere. Derivative TG curves (DTG) express the weight loss rate as a function of temperature.

### 3.8. ABTS·^+^ Radical Cation Scavenging Activity

The ABTS·^+^ radical cation scavenging activity was used to determine the antioxidant capacity of the extracts [58]. Briefly, 0.192 g of ABTS were dissolved in 50 mL of PBS at pH = 7.4 (for water-soluble extracts) or ethanol (organic-soluble extracts) and mixed with 33 mg of potassium persulphate overnight in the dark to yield the ABTS·^+^ radical cation. Prior to use in the assay, the ABTS·^+^ was diluted with either PBS or ethanol for an initial absorbance of ~0.70 ± 0.02 (1:50 ratio) at 734 nm, at room temperature. Free radical scavenging activity was assessed by mixing 1.0 mL diluted ABTS·^+^ with 10 µL of test antioxidant and monitoring the change in absorbance at 0, 1, 5, 10, 15 and 120 min (until a steady state was achieved). A calibration curve was built by using 6-Hydroxy-2,5,7,8-tetramethylchromane-2-carboxylic acid (Trolox). The antioxidant capacity of the extracts was expressed as µmol Trolox equivalents (TE)/g extract. All determinations were carried out in triplicate.

### 3.9. β-carotene–Linoleic Acid Assay

The antioxidant capacity of the extracts was also evaluated by the β-carotene–linoleic acid assay [59]. In brief, 4 mg of β-carotene was dissolved in 20 mL of chloroform. 2 mL of this solution were placed on a rotary evaporator and the chloroform was evaporated. Then, 50 µL of linoleic acid and 400 mg of Tween 40 were added and the content of the flask was mixed with stirring. After that, 100 mL of aerated distilled water was transferred to the flask and stirred vigorously. Five milliliters of the prepared β-carotene emulsion were transferred to a series of tubes containing 0.5 mL of each extract (0.5–5 mg/mL), BHT (0.1–1 mg/mL) as a positive control and ethanol as the negative control. The samples were incubated in a water bath at 50 °C for 120 min. The absorbance of each sample at 470 nm was measured every 15 min using a spectrophotometer. The E1 and E3 extracts were tested at 0.5–5 mg/mL, while lower concentrations of 0.5–3 mg/mL had to be used for the E2 extracts, due to their higher antioxidant capacity. All the determinations were carried out in triplicate.

### 3.10. Antifungal Activity Assays

*Penicillium digitatum* (Pers.:Fri.) Sacc. Pd1 (denoted as PDIP, and deposited in the Spanish Type Culture Collection with accession code CECT20795) was isolated from an infected grapefruit in Valencia. *Penicillium italicum* isolate PHI1 (denoted as PITC, CECT20909) was isolated from a mandarin stored at 4 °C in Valencia, Spain. *Penicillium expansum* Link isolate CMP-1 from Spain (denoted as PEX1, CECT20906) was isolated from a decayed ‘Golden’ apple after several months in storage in Lleida, Spain. *Geotrichum candidum* (isolate IATA 144) was isolated from an orange. *Botryitinia fuckeliana* (de Bary) Whetzel 1945 (*Botrytis cinerea* as anoamorph) and *Aspergillus niger* van Tieghem 1867 were obtained from the Spanish Type Culture Collection (CECT2100 and CECT2088, respectively). 

Conidial suspensions were prepared in sterile distilled water from a 7-day-old culture grown on potato dextrose agar (PDA) at 24 °C. The spore concentration was adjusted as required with the aid of a hemacytometer.

Growth inhibition assays were performed in 24-well microtitre plates (Nunc, Roskilde, Denmark) in a total volume of 800 μL of PDA containing serial dilutions of the extracts. Growth of strains was evaluated by depositing 5 μL of a conidial suspension (10^5^ conidia/mL) on each well. All samples were prepared in triplicate. Plates were incubated up to 7 days at 24 °C. Growth was determined by measuring the diameter of the colony using the software ImageJ. 

### 3.11. Antiviral Activity Assays

Feline calicivirus (FCV) F9 strain (ATCC VR-782) and murine norovirus (MNV) (kindly provided by Prof. H.W. Virgin, Washington University School of Medicine, USA) were assayed and propagated in CRFK (ATCC CCL-94) and RAW 264.7 cell lines (also provided by Prof. H.W. Virgin), respectively. Viruses were harvested and titrated as described by [60]. Briefly, infectious viruses were enumerated by determining the 50% tissue culture infectious dose (TCID_50_) with eight wells per dilution and 20 µL of inoculum per well using the Spearman–Karber method.

To elucidate the antiviral activity of *P. oceanica* extracts, 0.05 and 0.5 % of E1 US, E1 H_2_O, E2 US, E2 H_2_O, E3 US and E3 H_2_O extracts were incubated overnight with an equal volume of FCV or MNV suspensions (about ~7 log TCID_50_/mL) at 25 (FCV) or 37 °C (MNV). The samples were then diluted with 1.8 mL of Dulbecco’s Modified Eagle’s Medium (DMEM) supplemented with 10% fetal calf serum (FCS) and residual infectivity was determined by TCID_50_. Ten-fold dilutions of treated and untreated virus suspensions were inoculated into confluent in CRFK and RAW monolayers in 96-well plates. Then, infectious viruses were enumerated by cell culture assays as described above.

Each treatment was done in triplicate. Positive controls were virus suspensions added with PBS only under the same experimental conditions. The decay of FCV and MNV titres was calculated as log_10_ (N_x_/N_0_), where N_0_ is the infectious virus titre for untreated samples and Nx is the infectious virus titre for *P. oceanica*-treated samples.

### 3.12. Statistical Analysis

Data analysis was carried out using Statgraphics Stratus by Statgraphics Technologies, Inc. One-way analysis of variance (ANOVA) was done to determine the significant differences between sample means, at a significance level of *p* < 0.05. Mean comparisons were performed by the Tukey Test.

## 4. Conclusions

Water-soluble extracts from *Posidonia oceanica* waste biomass have been produced by means of the conventional organic solvent-based extraction method, as well as alternative greener methods based on heating and ultrasound-assisted methodologies from aqueous suspensions. An in-depth characterization of the generated extracts has been carried out to assess the differences in the composition and bioactivity of the extracts depending on the extraction method. The ethanol-soluble extracts (E1 H_2_O and E1 US) were mainly composed of minerals (72–75%), while the nonsoluble extracts (E2 H_2_O and E2 US) contained minerals (34–41%), proteins (27–37%) and carbohydrates (12–29%). On the other hand, the organic solvent-extracted fractions (E3 extracts) were mainly composed of lipids (56–79%). Specifically, the ultrasound treatment promoted the extraction of ashes and lipids, while greater amounts of carbohydrates, proteins and polyphenols were obtained with the heating extraction. The carbohydrate fraction in the E2 H_2_O extract was richer in galactose and glucose, while greater relative amounts of galacturonic acid and xylose were present in E2 US. 

Interestingly, all the extracts displayed remarkable antioxidant capacity values (higher than 200 µmol TE/g extract), as evidenced by the ABTS and β-carotene bleaching assays, which were not directly correlated to the phenolic contents, but rather seemed to be originated by the presence of some other compounds such as proteins and polysaccharides. Although the E3 extracts were the most effective ones reducing the growth of food-pathogen fungi such as *P. digitatum, B. cinerea* and *P. italicum*, the E2 extracts were capable of inhibiting the growth of *P. digitatum* and *B. cinerea*, being the E2 US more effective. In terms of antiviral activity, only the E3 extracts were able to reduce the infectivity of FCV and MNV in more than 2 log TCID_50_/mL.

These results show the potential of *Posidonia oceanica* waste biomass for the production of bioactive extracts with interest in food-related applications. Although bioactive compounds such as non-glycosylated polyphenols are more easily extracted by organic solvents, some other compounds such as polysaccharides, proteins and glycosylated polyphenols can be extracted by means of greener methodologies avoiding the use of toxic organic solvents. In particular, given their antioxidant and antifungal capacities, as well as their environmentally friendly character and ease of production, E2 H_2_O and E2 US extracts seem to be those with the most promising results, being both the hot water and ultrasound-assisted extractions promising alternatives to the conventional organic-solvent extraction methodology. Therefore, the potential of the alternative greener extraction methodologies has been demonstrated and a complete optimization of the processes to maximize extraction yields and bioactivity of the extracts will be carried out in the future.

## Figures and Tables

**Table 1:** Total carbohydrate, protein, lipid and phenolic content and antioxidant capacity of the extracts, evaluated by the ABTS and β-carotene bleaching methods. Different letters within the same column denote significant differences between extracts (*p* ≤ 0.05).

Extract	Carbohydrate Content (mg/g Extract)	Protein Content (mg BSA/g Extract)	Lipid Content (mg/g Extract)	Total Phenolics (mg GAE/g Extract)	Ash Content (mg/g Extract)	TEAC (µmol TE/g Extract) *	% β-Carotene Bleaching Inhibition ^†^
**E1 US**	12.2 ± 2.9 ^c^	58.4 ± 3.2 ^d^	n.d.	7.6 ± 2.2 ^c^	751.5 ± 1.4 ^a^	275.9 ± 10.2 ^c,d^	57.0 ± 2.3 ^c^
**E1 H_2_O**	12.9 ± 2.4 ^c^	70.9 ± 1.4 ^c^	n.d.	37.9 ± 8.0 ^b^	723.1 ± 6.6 ^b^	236.5 ± 16.5 ^e^	45.0 ± 1.8 ^d^
**E2 US**	117.7 ± 18.5 ^b^	269.3 ± 5.5 ^b^	n.d.	48.7 ± 5.5 ^b^	413.3 ± 8.8 ^c^	346.7 ± 5.6 ^b^	68.7 ± 4.2 ^b^
**E2 H_2_O**	287.1 ± 2.5 ^a^	363.0 ± 1.9 ^a^	n.d.	90.2 ± 17.6 ^a^	339.1 ± 9.5 ^d^	730.0 ± 12.2 ^a^	65.5 ± 5.3 ^b^
**E3 US**	-	-	785.3 ± 73.5 ^a^	79.6 ± 23.1 ^a^	180.7 ± 3.6 ^e^	211.2 ± 51.2 ^d,e^	70.8 ± 2.6 ^b^
**E3 H_2_O**	-	-	560.3 ± 85.3 ^b^	76.2 ± 3.3 ^a^	347.5 ± 22.9 ^d^	443.8 ± 150.2 ^b,c^	82.8 ± 3.5 ^a^

* TEAC values were calculated after 30 min. ^†^ Calculated for the extracts at a concentration of 0.5 mg/mL.

**Table 2:** Summary of the antifungal activity showed by all the different extracts tested in the following microorganisms at 3 and 7 dpi at their highest concentration (5 mg/mL). Antifungal activity was shown as the reduction in the diameter ± SD (%).

**Microorganism**	**E1 US**	**E1 H_2_O**	**E2 US**	**E2 H_2_O**	**E3 US**	**E3 H_2_O**
**3 dpi.**						
*Penicillium digitatum*	*-*	*-*	37 ± 9	12 ± 3	46 ± 2	14 ± 4
*Penicillium italicum*	*-*	*-*	-	-	40 ± 2	24 ± 2
*Penicillium expansum*	*-*	*-*	-	-	−(42 ± 3) ^a^	−(27 ± 3) ^a^
*Botrytis cinerea*	*-*	*-*	41 ± 12	19 ± 8	100	100
*Geotrichum candidum*	*-*	*-*	-	-	-	-
*Aspergillus niger*	*-*	*-*	-	-	−(100) ^a^	−(90 ± 4) ^a^
**6–7 dpi**						
*Penicillium digitatum*	*-*	*-*	24 ± 8	12 ± 4	-	-
*Penicillium italicum*	*-*	*-*	-	-	15 ± 3 ^b^	− ^b^
*Penicillium expansum*	*-*	*-*	-	-	−(22 ± 3) ^a^	−(19 ± 2) ^a^
*Botrytis cinerea*	*-*	*-*	-	-	61 ± 4	69 ± 5
*Geotrichum candidum*	*-*	*-*	-	-	-	-
*Aspergillus niger*	*-*	*-*	-	-	−(100) ^a^	−(70 ± 11) ^a^

^a^ Numbers between brackets show the % reduction in the sporulation. ^b^ With remarkable reduction in the hypha’s development (Figure S3-D).

**Table 3:** Sample codes for the different *P. oceanica* extracts.

Sample Code	Pre-Treatment	Extraction Method	Solvent	Fraction (Ethanol Precipitation)
**E1 US**	-	Ultrasound	Water	Supernatant
**E1 H_2_O**	-	Heating	Water	Supernatant
**E2 US**	-	Ultrasound	Water	Precipitate
**E2 H_2_O**	-	Heating	Water	Precipitate
**E3 US**	Ultrasound	Soxhlet	Toluene:ethanol	-
**E3 H_2_O**	Heating	Soxhlet	Toluene:ethanol	-

## Supplementary Materials

The following are available online at https://www.mdpi.com/1660-3397/17/7/409/s1, Figure S1: Derivative thermogravimetric (DTG) curves of *P. oceanica* extracts obtained by water-based extractions (A) and organic solvent-based extractions (B). Figure S2: β-carotene bleaching inhibitory activity of the *P. oceanica* extracts tested at different concentrations. (A) E1, (B) E2 and (C) E3. Figure S3: Antifungal activity of E2 extracts vs. *P. digitatum* (A) and E3 vs. *B. cinerea* (B) at 3 days postinoculation, and E3 vs. *P. italicum* (C) and *A. niger* (D) at 7 days postinoculation.
