# Supplementary material for: In-Depth Characterization of Bioactive Extracts from Posidonia oceanica Waste Biomass
Source: Mar Drugs. 2019 Jul 9;17(7):409. doi: 10.3390/md17070409 (PMC6669500; doi:10.3390/md17070409)
Supplement: Supplementary file 1 [file marinedrugs-17-00409-s001.pdf]

In-depth Characterization of Bioactive Extracts from *Posidonia oceanica* Waste Biomass

Isaac Benito-González [1], Amparo López-Rubio [1], Antonio Martínez-Abad [2], Ana-Rosa Ballester [3], Irene Falcó [1,4], Luis González-Candelas [3], Gloria Sánchez [1], Jesús Lozano-Sánchez [5,6], Isabel Borrás-Linares [5], Antonio Segura-Carretero [5,7] and Marta Martínez-Sanz [1,*]

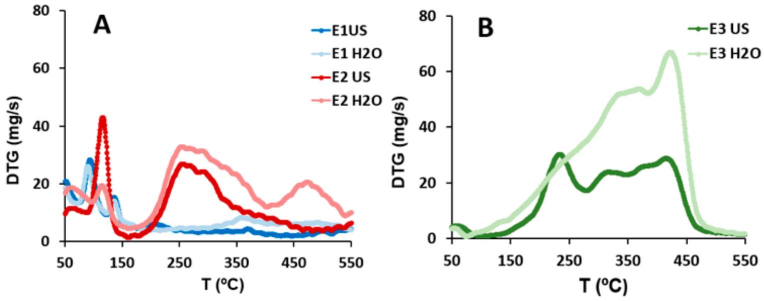

Figure S1. Derivative thermogravimetric (DTG) curves of *P. oceanica* extracts obtained by water-based extractions (A) and organic solvent-based extractions (B).

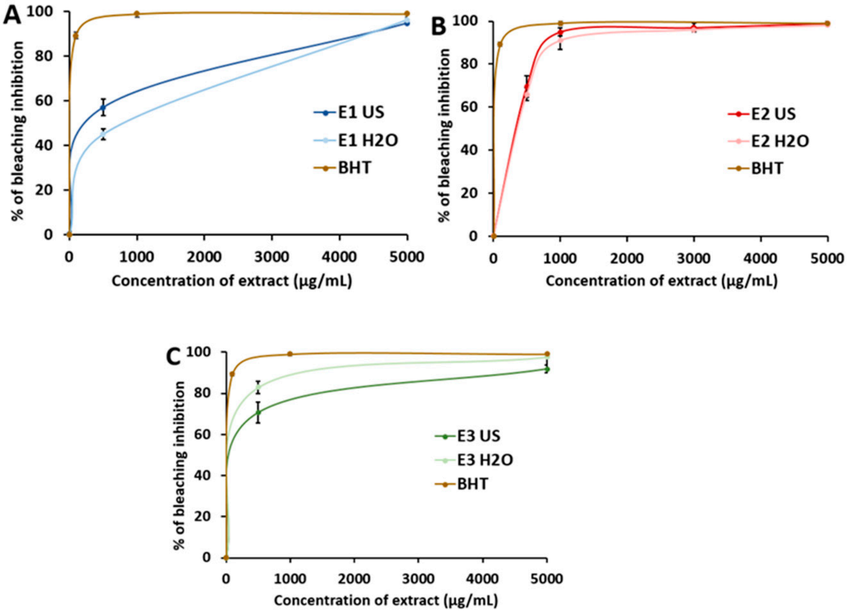

Figure S2. β-carotene bleaching inhibitory activity of the *P. oceanica* extracts tested at different concentrations. (A) E1, (B) E2 and (C) E3.

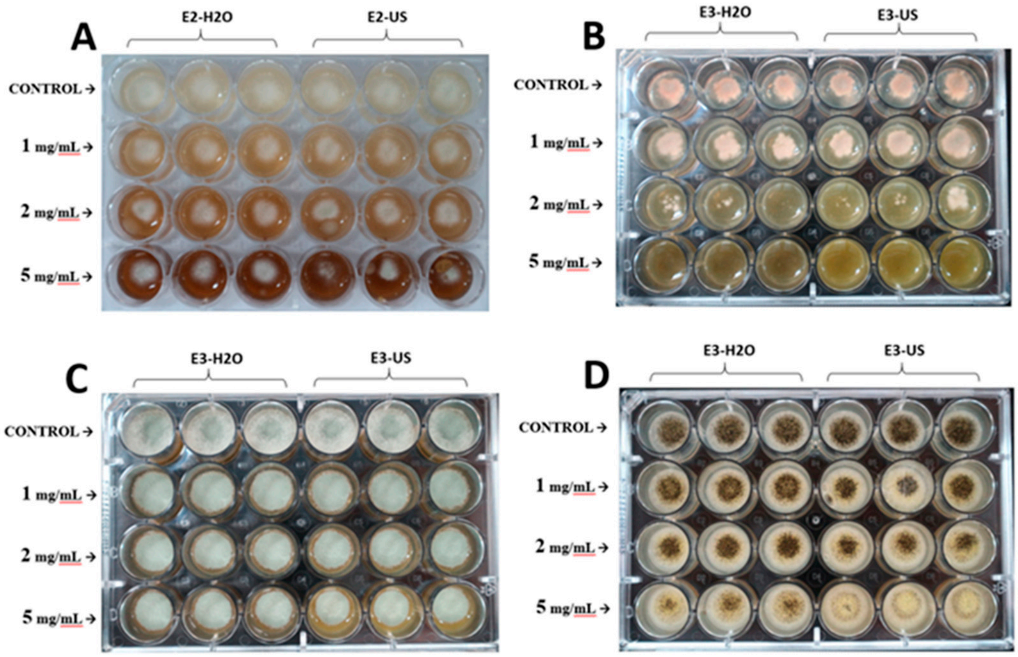

Figure S3. Antifungal activity of E2 extracts vs. *P. digitatum* (A) and E3 vs. *B. cinerea* (B) at 3 days post inoculation, and E3 vs. *P. italicum* (C) and *A. niger* (D) at 7 days post inoculation.
